# Internal Lamellar and Inverse Hexagonal Liquid Crystalline Phases During the Digestion of Krill and Astaxanthin Oil-in-Water Emulsions

**DOI:** 10.3389/fbioe.2019.00384

**Published:** 2019-12-05

**Authors:** Anan Yaghmur, Saleh Lotfi, Sarah Atoussa Ariabod, Gizem Bor, Mark Gontsarik, Stefan Salentinig

**Affiliations:** ^1^Department of Pharmacy, Faculty of Health and Medical Sciences, University of Copenhagen, Copenhagen, Denmark; ^2^Laboratory for Biointerfaces, Empa, Swiss Federal Laboratories for Materials Science and Technology, St. Gallen, Switzerland; ^3^Department of Chemistry, University of Fribourg, Fribourg, Switzerland

**Keywords:** astaxanthin oil, krill oil, lamellar and inverse hexagonal liquid crystalline phases, lipolysis, omega-3 polyunsaturated fatty acids, synchrotron small angle X-ray scattering

## Abstract

Krill oil represents an important alternative natural source of omega-3 (*ω-3*) polyunsaturated fatty acids (PUFAs). Considering the beneficial health effects of these essential fatty acids, particularly in various disorders including cancer, cardiovascular, and inflammation diseases, it is of paramount importance to gain insight into the digestibility of krill oil. In this work, we study the fate of krill oil-in-water emulsion, stabilized by sodium caseinate, during lipolysis by coupling time-resolved synchrotron small-angle X-ray scattering (SAXS) to flow-through lipolysis model. For gaining further insight into the effect of *ω-3* PUFA-containing oil type on the dynamic structural features occurring during lipolysis, two additional astaxanthin oil-in-water emulsions, stabilized using either sodium caseinate or citrem, were subjected to lipolysis under identical experimental conditions. In addition to the difference in lipid composition in both oils, *ω-3* PUFAs in astaxanthin oil, similar to fish oil, exist in the form of triacylglycerols; whereas most of those in krill oil are bound to phospholipids. SAXS showed the formation of highly ordered nanostructures on exposure of these food emulsions to the lipolysis medium: the detection of a biphasic feature of coexisting inverse hexagonal (H_2_) and lamellar (L_α_) liquid crystalline phases in the digested krill oil droplets' interiors, as compared to a neat L_α_ phase in the digested astaxanthin oil droplets. We discuss the dynamic phase behavior and describe the suggested important role of these phases in facilitating the delivery of nutrients throughout the body. In addition, the potential implication in the development of food and drug nanocarriers is briefly described.

## Introduction

**Figure 1:**
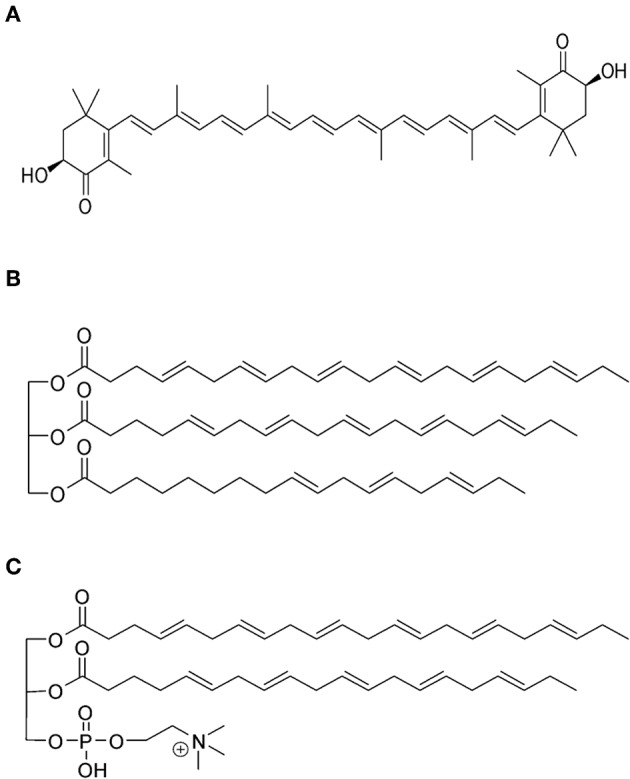
**(A)** The molecular structure of the natural fat-soluble antioxidant astaxanthin that provides KO with the blood reddish color. **(B,C)** illustrate the main difference in the *ω-3* PUFA composition between FO and KO. In **(B)**, an example on the typical presence of the *ω-3* PUFAs docosahexaenoic acid (DHA; 22:6 n-3) and eicosapentaenoic acid (EPA; 20:5 n-3) in the form of triacylglycerols is given; whereas **(C)** shows that these ω-3 PUFAs are typically conjugated to the glycerol moieties of phospholipids in KO. These molecular structures were drawn with ChemBioDraw Ultra version 12 (PerkinElmer Informatics, Waltham, MA, USA).

Fish (FO) and krill (KO) oils are important natural sources of omega-3 (*ω-3*) polyunsaturated fatty acids (PUFAs). Mackerel, salmon, and herring are the main sources of marine *ω-3* PUFAs; whereas KO is extracted from the Antarctic shrimp-like crustacean *Euphausia superba*. This Krill lives in the deep ocean and itself is a food source for many bigger marine animals (e.g., whales and seals) (Fragakis, 2008; Atkinson et al., 2009). KO is Generally Recognized as Safe (GRAS) by the American Food and Drug Administration (FDA), and secures an unconventional food source for *ω-3* PUFAs. It is rich in *ω-3* PUFA-containing phospholipids (Ramprasath et al., 2013). In addition, it is rich in different compounds that secure its protection against oxidation such as vitamins A and E, and the carotenoid astaxanthin that has typical concentrations of 200–400 ppm (Kolakowska et al., 1994; Atkinson et al., 2009; Ramprasath et al., 2013). Figure 1A presents the molecular structure of astaxanthin. The most abundant phospholipid in KO is phosphatidylcholine (PC), and the deep red color of this oil is attributed to the presence of astaxanthin. In addition to phospholipids (19–81%) and triacylglycerols (12–30%), KO contains the following amphiphilic compounds: free fatty acids (FFAs), cholesterol, and diacylglycerols. The attractiveness of KO as an important source of natural nutritional compounds relies also on the published preclinical and clinical investigations on its safety, well-tolerance, and lack of adverse effects (Ruggiero-Lopez et al., 1994; Sampalis et al., 2003; Bunea et al., 2004; Deutsch, 2007; Maki et al., 2009).

The dietary intake of FO and KO is important and associated with promoting positive health effects. For instance, it was found that FO intake lowers the blood lipid concentration and reduces systolic blood pressure (Araujo et al., 2014); whereas KO promotes lipid metabolism, controls hyperlipidemia, and offers a greater potential than other marine oils, owing particularly to its richness with phospholipids. It was found that KO exerts anti-inflammatory and hypolipidemic effects (Ulven and Holven, 2015; Ristić-Medić et al., 2018). It is also more effective at lowering cholesterol levels than FO (Massrieh, 2008). It was also recently reported using randomized controlled human trials with 36 healthy adults that this oil intake is associated with a significant reduction in blood glucose concentration (Rundblad et al., 2018). Another important finding with potential health implications, as recently suggested by Bruheim et al. (2015), is the effectiveness of KO in the treatment of obesity, which is one of the major modern health problems.

As illustrated in Figures 1B,C, an important difference in the *ω-3* PUFA composition between FO and KO is the presence of a large fraction (30–65%) of fatty acids conjugated to the glycerol moieties of phospholipids in KO; whereas the rest exists in the form of triacylglycerols (Ramprasath et al., 2015). In FO, *ω-3* PUFAs are mainly present in the form of triacylglycerols (Ramprasath et al., 2015; Ulven and Holven, 2015). It was proposed that binding *ω-3* PUFAs to phospholipids facilitates their incorporation into cell membranes (Bunea et al., 2004; Maki et al., 2009; Schuchardt et al., 2011; Ulven et al., 2011; Ramprasath et al., 2013). In addition, it provides self-assembling properties to KO, enhances the bioavailability, and improves the oxidative stability as compared to FO (Henna Lu et al., 2011; Ulven et al., 2011; Wu et al., 2016).

*ω-3* PUFAs in KO and FO, particularly eicosapentaenoic (EPA; 20:5, n-3) and docosahexaenoic (DHA; 22:6, n-3) acids, are important nutritional compounds and have beneficial health effects in various disorders in humans including inflammation diseases, cancer, and cardiovascular diseases, among others (Bradberry and Hilleman, 2013; Wang et al., 2014; Richter et al., 2016; Yaghmur et al., 2017). EPA and DHA should be integrated in our diet as humans cannot synthesize these essential fatty acids or produce their precursor α-linolenic acid (Davidson et al., 2012; Swanson et al., 2012; Kohler et al., 2015; Ramprasath et al., 2015; Mori, 2018). As compared to the triacylglycerol form of EPA and DHA in FO, the superior bioavailability of KO is most likely attributed to the presence of these essential acids in the phospholipid-enriched form: 70 % of EPA and DHA are bound to phospholipids in KO (Bunea et al., 2004; Kutzner et al., 2017). It was suggested that the amphiphilic compounds (mainly phospholipids) in KO play an important role in enhancing the absorption and intestinal digestion (Davidson et al., 2012; van Hoogevest and Wendel, 2014; Ulven and Holven, 2015).

Similar to FO, recent reports focus on improving further the bioavailability and enhancing the oxidation stability of KO by its solubilization in food-grade lipid nanocarriers, mainly emulsions and self-emulsifying drug delivery systems (Neslihan Gursoy and Benita, 2004; Salminen et al., 2013; Bonaterra et al., 2017; Seto et al., 2018). However, the number of studies is still limited in the literature and further investigations are still needed to fully characterize these nanocarriers and gain further insight into their colloidal and oxidation stabilities under different experimental conditions.

Previous studies reported that emulsification of FO prior to digestion enhances the bioavailability and absorption of *ω-3* PUFAs including EPA and DHA as compared to the corresponding bulk (non-dispersed) oils (Armand et al., 1994; Borel et al., 2001). In case of colloidal dispersions including food emulsions, the competitive adsorption behavior in the stomach among the emulsified oils, lipids, and other food bioactive molecules such as enzymes, proteins, and polysaccharides is associated with the tendency of substances with higher surface activity to be accommodated at the interface (McClements et al., 2009). The triglyceride composition was also found to affect the size distribution, the surface charge, and the nanostructure of digested colloidal dispersions (Zhang et al., 2015). In these investigations, an important aspect that should be taken into account is related to the dynamic changes in the structural and morphological features, size characteristics, and composition of food emulsion droplets, and related colloidal nanoparticles (e.g., liposomes, cubosomes, and hexosomes), on their way through the gastro intestinal tract (Zhang and McClements, 2018).

In previous reports, the nanostructure formation during the digestion of triglyceride-based emulsions including milk, mayonnaise, and a model triolein-in-water emulsion was investigated (Patton and Carey, 1979; Salentinig et al., 2011, 2015a, 2017; Marze et al., 2015; Salentinig, 2019; Vithani et al., 2019). The major part of lipid digestion takes place in the small intestine by pancreatic lipase and various cofactors (colipase, bile salts, and calcium) at typical pH in the range of 6.0–8.0 (Golding and Wooster, 2010). In this process, pancreatic lipase adsorbs onto the emulsion droplets and stereospecifically hydrolyzes the two outer ester bonds of the triglycerides, denoted as *sn-1* and *sn-3*, resulting in the generation of the *sn-2* monoglyceride and the corresponding free fatty acids (FFAs) (Salentinig et al., 2015b). This leads to structural alterations in the emulsified oil droplets' interiors: the occurrence of colloidal objects with self-assembled interiors including inverse non-lamellar liquid crystalline structures and micellar phases (Salentinig et al., 2011, 2015a, 2017), similar to the nanostructural features of cubosomes, hexosomes, and other related colloidal nanodispersions (Yaghmur and Glatter, 2009; Azmi et al., 2015; Meli et al., 2015; Yaghmur et al., 2017, 2019; Shao et al., 2018). It was proposed that these nano-self-assemblies act as structured nanocarriers for facilitating transport and absorption of poorly water-soluble nutrients (Huang et al., 2010; Nik et al., 2011; Luo et al., 2012; Teng et al., 2013; Guttoff et al., 2015; Prajapati et al., 2019a). In recent studies, there is a growing interest in the development of such nanocarriers, mainly cubosomes and hexosomes, for drug-delivery and bio-imaging applications (Nilsson et al., 2013; Azmi et al., 2015, 2016, 2018; Meli et al., 2015; Wibroe et al., 2015; Gontsarik et al., 2016, 2019; Angelova et al., 2017; Bor et al., 2019; Prajapati et al., 2019b). In the development of these non-lamellar liquid crystalline nanoparticles, the most investigated monounsaturated monoglyceride is monoolein (Nakano et al., 2002; Yaghmur and Glatter, 2009). The possible use of new *ω-3* PUFA monoglycerides as main lipid constituents in the development of these nanocarriers for the delivery of therapeutic agents, nutraceuticals, and their combinations was recently discussed (Yaghmur et al., 2017; Shao et al., 2018).

**Figure 2:**
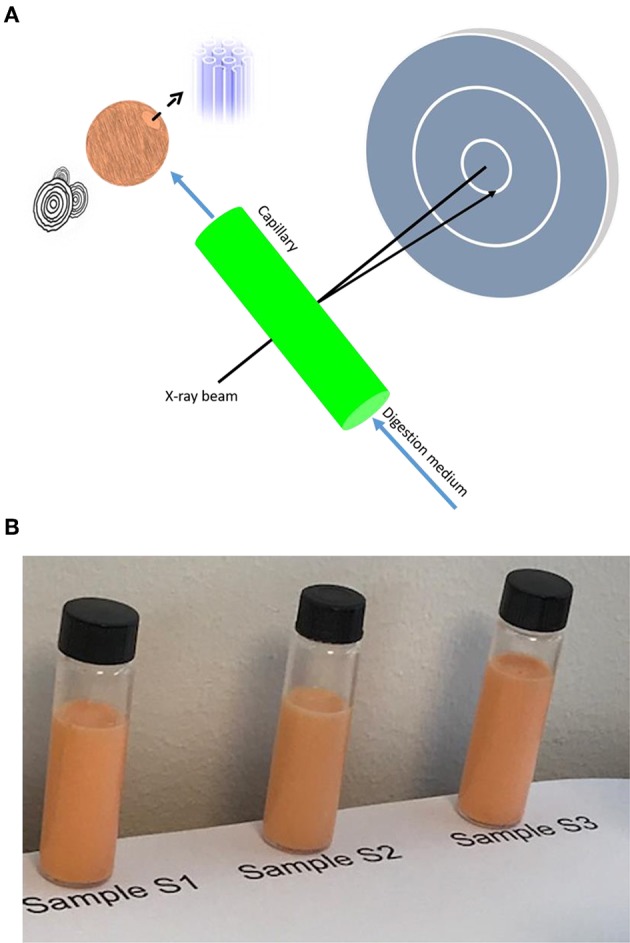
**(A)** Schematic illustration of the flow-through lipolysis model combined with synchrotron SAXS for investigating the colloidal behavior of krill oil (KO) and astaxanthin oil (AO) emulsions during *in vitro* digestion. In this set-up, the lipolysis vessel was connected to a flow-through 1.5 mm quartz capillary and pH was maintained at 6.5. SAXS findings indicated the formation of liquid crystalline nanostructures on exposure of these *ω-3* PUFA-containing emulsions to the lipolysis medium. **(B)** Pictures taken for the three investigated samples (S1–S3) after 3 months of preparation and storage at room temperature.

In the present work, it was the main interest to combine synchrotron small-angle X-ray scattering (SAXS) with flow through lipolysis model for investigating the colloidal behavior of krill oil (KO) and astaxanthin oil (AO) emulsions during digestion. Herein, it should be emphasized that synchrotron SAXS is a powerful tool for dynamic structural investigations and provides important information on structural alterations in these emulsion droplets during the digestion process. It is also important to emphasize that this research on the *in situ* dynamic formation of lamellar and non-lamellar structures during digestion of food emulsions is still in its infancy and there is an urgent need for further fundamental research studies to translate the experimental findings to relevant future food and pharmaceutical applications. Figure 2A schematically illustrate the applied set-up. In contrast to KO, *ω-3* PUFAs in AO, which is produced from krill harvested in the antarctic ocean, are mainly present in the form of triacylglycerols. Hence, KO will be mainly digested by phospholipase and AO by pancreatic lipase in the small intestine. This study aims at comparing the digestion behavior of these two important sources of *ω-3* PUFAs and linking the generated nanostructures to their main lipid constituents. These investigations are important for gaining insight into the dynamic structural transitions occurring due to variations in the lipid composition of digested KO and AO emulsion droplets during lipolysis. As lipase is a surface-active enzyme and needs to access the lipid-water interface for initiating digestion, the stabilizer type may have a major impact on this process. To further shed light on the effect of the stabilizer type on digestion, we compared the dynamic structural features of AO emulsion stabilized by sodium caseinate from bovine milk with that stabilized using the anionic food-grade emulsifier citrem.

Taking into account the health beneficial effects of *ω-3* PUFAs, the lipolysis-mediated generation of nanoparticles enveloping an internal liquid crystalline nanostructure, similar to cubosomes, hexosomes, and related nanoparticles during the digestion of model *ω-3* PUFA emulsions may have implications in relation to soft self-assembled nanocarrier design for delivery of *ω-3* PUFA alone or in combination with drugs or other nutraceuticals. We should bear in mind that the potential translation of the experimental findings to future functional food and drug delivery applications requires combining SAXS investigations with relevant biological evaluations, and further research efforts on the influence of the digestive conditions on size characteristics and morphological features of the generated particles. The implications and potential future applications of such colloidal nanocarriers for drug and food delivery applications are discussed in a recent review (Salentinig, 2019).

## Materials and Methods

### Materials

Krill (KO, Superba^TM^ 2) and astaxanthin (AO, Qrill^TM^) oils were kindly donated by Aker Biomarine Manufacturing LLC in Houston (Texas, US) and Aker biomarine Antarctic (Lysaker, Norway), respectively. According to the supplier's specifications, the total phospholipid content of KO was about 48.0 wt% comprising phosphatidylcholine (about 44.0 wt%) and choline (about 6.0 wt%). It has a total *ω-3* PUFA content of 24.2 wt% including EPA (14.8 wt%) and DHA (6.7 wt%) (Table 1). In addition, it contains a residual amount of omega-6 (ω*-6*) fatty acids (about 1.0 wt%) and about 287 (μg/g oil) astaxanthin. AO contains a greater concentration of astaxanthin (≥750 μg/g oil), and consists of about 16.8 wt% *ω-3* PUFAs, and about 0.5 wt% free fatty acids (FFAs) (Table 1). Among *ω-3* PUFAs, AO contains about 2.8 wt% DHA and about 4.3 wt% EPA (Table 1). According to the supplier's specifications, *ω-3* PUFAs exist in the form of triacylglycerols in AO. The food emulsifier sodium caseinate from bovine milk, and sodium azide (SA) that was used as an antimicrobial preservative were purchased from Sigma-Aldrich (St. Louis, USA). Grinsted® citrem LR10, a commercial anionic food emulsifier made from sunflower oil, was received as a gift from Danisco A/S (Copenhagen, Denmark). It consists of citric acid esters of mono- and di-glycerides, where oleic acid content is 79.1% of its total fatty acid content (Amara et al., 2014). For producing KO and AO emulsions, 67 mM phosphate buffer (PBS) at pH 7.40 was prepared by using Dulbecco's saline tablets purchased from Sigma Aldrich (St. Louis, Missouri, USA). In the *in vitro* digestion study, pH was adjusted to 6.5 using either 0.2 M NaOH or 0.2 M HCl (p.a. grade, Sigma-Aldrich, St. Louis, MO, USA). Pancreatin from porcine pancreas (8 × USP grade pancreatin activity) was purchased from Sigma-Aldrich (St. Louis, MO, USA). It is a commercial mixture of digestive enzymes produced by the exocrine cells of the pancreas and contains components such as lipase, amylase, trypsin, ribonuclease, and protease (Salentinig et al., 2017). For preparing the buffer, Milli-Q water was collected from Millipore Direct-Q3 UV system (Billerica, MA, USA). All materials were used without further purification.

**Table 1:** Average composition of astaxanthin oil (AO) and Krill oil (KO).

**Content**	**AO**	**KO**
**Lipids**	**(wt%)**	**(wt%)**
Total *ω-3* PUFAs	16.8	24.2
EPA	4.3	14.8
DHA	2.8	6.7
Total phospholipids		48.0
Phosphatidylcholine (PC)	<1.0	44.0
Choline		6.0
Total *ω-6* PUFAs		<3
Triacylglycerols	96.0	<34
Diacylglycerols	2.0	
Monoacylglycerols	<1.0	
Free fatty acids (FFAs)	0.5	
Total neutral lipid	99.8	
Astaxanthin	≥750 μg/g oil	287 μg/g oil

### Preparation of KO and AO Emulsions

Three oil-in-water (O/W) emulsions stabilized using sodium caseinate (samples S1 and S2) or citrem (sample S3) were prepared by means of ultrasonication (Ultrasonic Processor Qsonica 500, Qsonica LLC, Newtown, CT, USA) for 5 min in pulse mode (5 s pulses interrupted by 2 s breaks) at 30% of its maximum power (500 W). For the preparation of these samples, the solutions of binary mixtures of KO/sodium caseinate, AO/sodium caseinate, and AO/citrem were prepared at room temperature at a fixed KO (or AO)/sodium caseinate (or citrem) weight ratio of 4:1 and dispersed in excess PBS with pH of 7.4. They were composed of 5.0 wt% binary oil/emulsifier mixture of KO/sodium caseinate (sample S1), AO/sodium caseinate (sample S2), and AO/citrem (sample S3) and a small amount of sodium azide (0.01 wt%), that was used as an antimicrobial preservative. In addition, they were flushed with nitrogen gas for 2 min, capped, covered by aluminum foil, and incubated at room temperature prior to the planned investigations.

### Zeta Potential and Droplet Size Measurements

The zeta potentials and the mean hydrodynamic diameters were measured using Zetasizer Nano ZS (Malvern Instruments, Worcestershire, UK) equipped with a 633 nm laser and 173° detection optics. The measurements were performed at room temperature on samples diluted 100x in 67 mM PBS, and data acquisition and analysis were conducted using Malvern DTS v.6.34 software (Malvern instruments, Worcestershire, UK). For the viscosity and refractive index, the values of pure water were used.

### Lipolysis Model

The *in vitro* digestion was studied using online synchrotron SAXS similar to our previous studies (Salentinig et al., 2011, 2015a, 2017). The digestion reaction was performed at 37°C in a thermostated 20 mL reaction vessel under constant magnetic stirring. This vessel was connected to a flow-through 1.5 mm quartz capillary using HPLC tubing and pH was maintained at 6.5 in all experiments with an autotitrator (Titrando 906, Metrohm AG, Herisau, Switzerland). In the small-angle X-ray scattering (SAXS) set-up, the capillary was placed in the capillary holder and the digestion medium was circulated using a peristaltic pump, while recording SAXS in real time for monitoring the evolution of the self-assembled nanostructure during the sample digestion.

Five milliliters of sample (emulsion) was mixed with 4 mL of buffer at pH 6.5 and circulated during the real-time SAXS measurements. In this study, the pancreatin solution was prepared by mixing 5 g pancreatin extract into 20 mL of buffer at pH 6.5 and stirring using a magnetic stirrer for 30 min at 4°C. The mixture was then centrifuged at 5,000 xg for 20 min at 4°C, and the supernatant filled into an 1 mL syringe. The digestion reaction was started by using a syringe pump (neMesys, centoni GmbH, Korbußen, Germany) for remotely adding 1 mL of the prepared pancreatin solution with a speed of approximately 0.2 mL/s. Then, the pH-stat titrated the digestion mixture with 0.2 M NaOH solution in order to maintain pH at 6.5.

### Synchrotron Small-Angle X-Ray Scattering (SAXS)

For gaining insight into the effect of *in vitro* digestion on the structural features of the three prepared oil-in-water emulsions, the lipolysis model was combined with synchrotron SAXS. The measurements were performed on the cSAXS beamline at the Swiss Light Source, Paul Scherrer Institute (PSI) (Villigen, Switzerland). An X-ray beam having a wavelength of 1.107 Å at X-ray energy of 11.2 keV was used. The sample-detector distance was 2152.4 mm and the scattering vector, *q*, covered a range of 0.1–5.0 nm^−1^ (*q* = *4*π*/*λ *sin*θ, where λ is the wavelength and *2*θ is the scattering angle). All experiments were done at 37°C (±0.1°C). The 2D SAXS patterns were acquired over 3,500 s with an exposure time of 1 s with 9 s delay between frames, using an Pilatus 2M detector (in-house prototype) with an active area of 254 × 289 mm^2^ and a pixel size of 172 x 172 μm^2^. The 2D scattering frames were radially integrated into one-dimensional (1-D) curves and plotted as a function of relative intensity *I(q)* vs. *q*. After subtracting the background scattering (PBS buffer), all Bragg peaks were fitted by Lorentzian distributions. The lattice parameter (*a*) and *d*-spacing of the inverse hexagonal (H_2_) and lamellar (L_a_) liquid crystalline nanostructures, respectively, were gleaned from SAXS reflections.

## Results and Discussion

### Size and Surface Charge Characteristics of KO and AO Emulsions

The mean droplet diameters for sodium caseinate-stabilized KO and AO emulsions (samples S1 and S2) were 294.6 ± 22.7 and 296.8 ± 25.8 nm, respectively, and consistent with those previously reported for KO dispersions prepared in the presence of lysolecithin, glycerin, and hydroxypropyl methylcellulose (Seto et al., 2018). The polydispersity indices (PDIs) for both samples were 0.26 ± 0.08 and 0.20 ± 0.11, respectively (Table 2). The presence of marine phospholipids in KO, and natural emulsifiers including diacylgylcerols, monoacylglycerols (monoglycerides), phospholipids, and FFAs in AO improves the emulsifying properties of these oils, and enhances, therefore, the colloidal stabilization of the prepared oil-in-water (O/W) emulsions. For citrem-stabilized AO emulsion (sample S3), the use of the anionic emulsifier citrem instead of sodium caseinate was associated with a decrease in the mean droplet size and PDI to 226.4 ± 7.9 nm and 0.152 ± 0.07, respectively. The droplets in the three samples were negatively charged based on zeta potential measurements (Table 2). Clearly, the replacement of sodium caseinate by citrem led to a decrease in zeta potential from −19.85 ± 1.49 to −31.8 ± 1.86 mV owing to the adsorption of the negatively charged citrem molecules into the outer surfaces of AO droplets. In literature, it was reported that a good colloidal stability for ionic aqueous dispersions including emulsions can be achieved in the presence of single charged emulsifiers such as citrem on dispersing droplets with zeta potential values around ±30 mV in excess water (Honary and Zahir, 2013). In addition to its use as stabilizer with an anti-oxidative property for various food emulsions (Gudipati et al., 2010; Berton et al., 2011), citrem is attractive for use as a safe emulsifier for stabilizing lamellar and non-lamellar liquid crystalline nanoparticles owing to its hemocompatiblity and poor activation of the complement system (Nilsson et al., 2012; Hedegaard et al., 2013; Wibroe et al., 2015; Prajapati et al., 2018). In our study, the three investigated samples were visually inspected and found to be colloidally stable at room temperature for at least 3 months after preparation, see images in Figure 2B.

**Table 2:** Size characteristics and zeta potential values for KO-in-water and AO-in-water emulsions stabilized by using either sodium caseinate from bovine milk or citrem.

**Sample**	**ζ-potential (mV)**	**Mean size (diameter, nm)**	**PDI**
S1: KO-in-water emulsion^a^	−9.29 ± 0.41 mV	294.6 ± 22.7	0.26 ± 0.08
S2: AO-in-water emulsion^a^	−19.85 ± 1.49 mV	296.8 ± 25.8	0.20 ± 0.11
S3: AO-in-water emulsion^b^	−31.8 ± 1.86 mV	226.4 ± 7.9	0.15 ± 0.07

*These samples were prepared using PBS at pH 7.4*.

a *The emulsions (samples S1 and S2) were stabilized using sodium caseinate at KO (or AO)/sodium caseinate weight ratio of 4:1*.

b *The emulsion (samples S3) was stabilized using citrem at AO/citrem weight ratio of 4:1*.

### Digestion Triggered Dynamic Structural Alterations in KO and AO Emulsions

**Figure 3:**
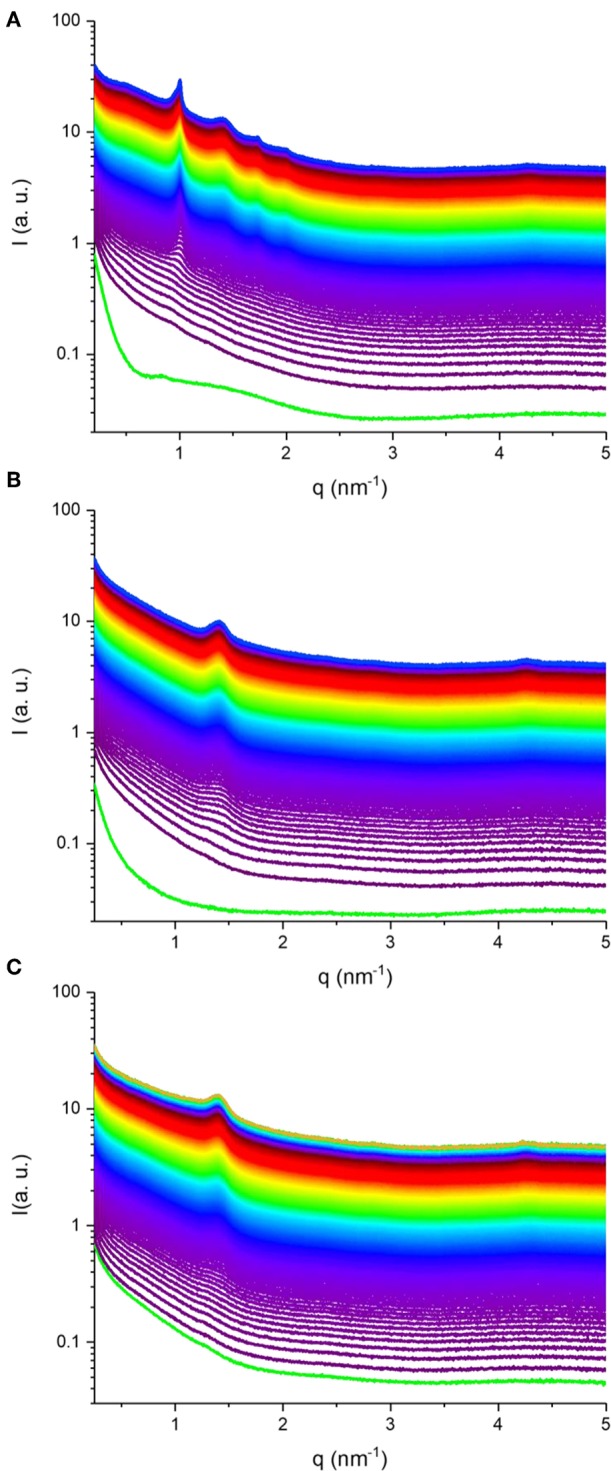
SAXS patterns for three *in situ* digested KO emulsion **(A)**, and AO emulsions stabilized by using sodium caseinate **(B)** and citrem **(C)**. In **(A–C)**, the time-resolved SAXS experiments for about 49, 50, and 57 min, respectively, were conducted on the three investigated KO and AO emulsions (samples S1–S3, see Table 2) at 37°C. The green SAXS patterns in **(A,B)** are taken for the two KO **(A)** and AO **(B)** emulsions stabilized by using sodium caseinate emulsions before the addition of pancreatin extract containing the lipase. For the *in situ* SAXS experiments, the first SAXS pattern was plotted 20 s after addition of the pancreatin extract, and then SAXS patterns were plotted every 10 s during the lipolysis process.

**Figure 4:**
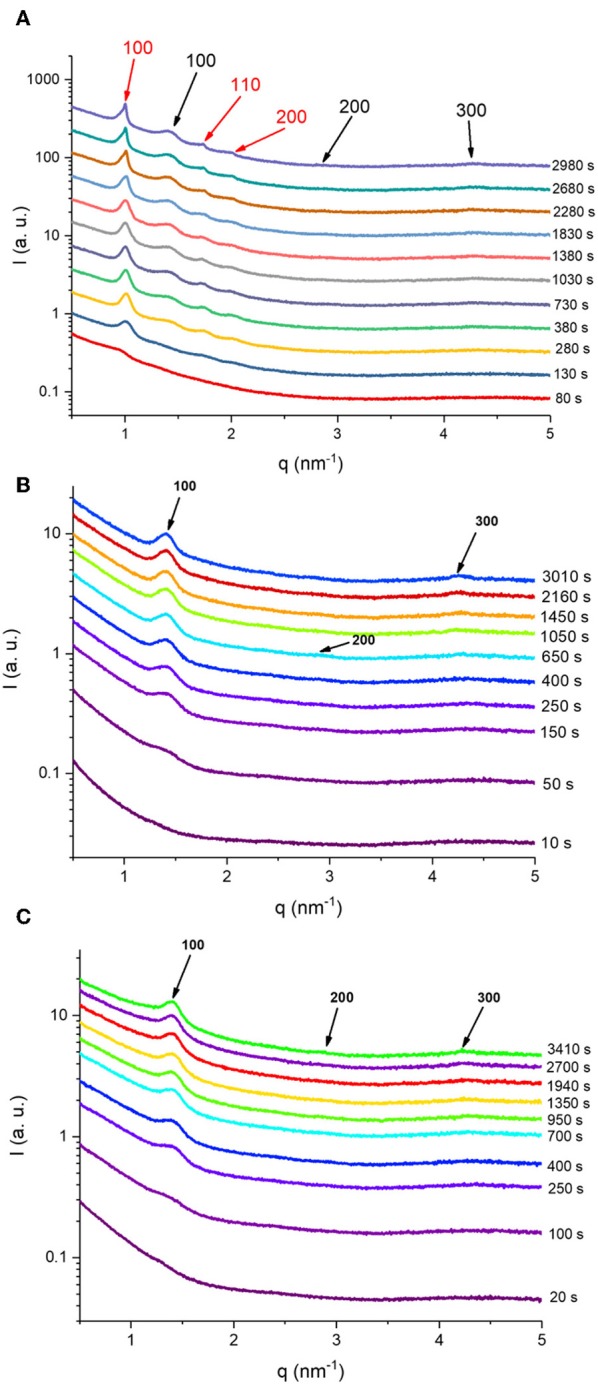
Selected SAXS patterns are presented at different elapsed times for the three *in situ* digested KO emulsion **(A)**, and AO emulsions stabilized by using sodium caseinate **(B)** and citrem **(C)**. The Bragg peaks are represented with red and black colors for the corresponding Miller indices of the inverse hexagonal (H_2_) and lamellar (L_α_) phases, respectively. The intensities in **(A–C)** have been shifted by a constant arbitrary factor for better visibility.

Synchrotron SAXS coupled with flow-through lipolysis model was used to gain insight into the fate of KO and AO emulsion droplets under simulated conditions of the small intestine, which is considered the main site for lipid digestion that leads to structural alterations or generation of nano-self-assemblies (Salentinig et al., 2015a, 2017; Salentinig, 2019; Vithani et al., 2019). At pH of 6.5, where pancreatic lipase has its maximum activity, the digestion-triggering dynamic structural alterations in the three KO and AO emulsions (samples S1–S3) were investigated. Figure 3 presents the time-resolved SAXS intensity profiles for the digested KO and OA emulsions. Before the addition of pancreatin extract containing the lipase, a weak reflection at *q* of about 0.83 nm^−1^ can be observed in the SAXS pattern of the KO emulsion (Figure 3A), that is absent in the AO systems (Figures 3B,C). This reflection may arise from the formation of traces of multi-lamellar structures during emulsification of KO that contains up to 48.0 wt% phospholipids. Representative SAXS diffraction patterns at different elapsed times of lipolysis are also presented in Figure 4. Before the addition of pancreatin extract, the detected upturn of the SAXS patterns of the three investigated emulsions, as shown in Figure 3, at relatively low *q* values is most likely attributed to scattering from emulsion droplets with dimensions larger than the resolution of the SAXS setup. For these emulsions, it was evident from the SAXS patterns presented in Figures 3, 4 that KO and AO digestion was associated with the appearance of either a biphasic feature of a lamellar (L_α_) phase coexisting with an inverse hexagonal (H_2_) phase (Figures 3A, 4A) or a neat L_α_ phase (Figures 3B,C, 4B,C). Depending on the lipid structure (e.g., unsaturation degree, acyl chain length, and structure of polar headgroup) and composition, the trends in appearance and evolving behavior of the lyotropic lamellar and non-lamellar liquid crystalline phases during lipolysis in these emulsion droplets were consistent with previous investigations on the *in situ* lipolysis of triolein emulsions, milk, and triglyceride-containing emulsified food products such as mayonnaise (Salentinig et al., 2011, 2015a, 2017) (Table 2). The rapid occurrence of self-assembled interiors in these KO and AO emulsion droplets (Figure 3) is attributed to the lipolysis-mediated generation of amphiphilic lipids and the involved fast molecular re-distribution and alterations in the lipid composition. Referring back to the lipolysis products, the adsorption of pancreatic lipase into the emulsion droplets' surfaces enhances the hydrolysis of triglycerides resulting in monoglycerides, diglycerides, and FFAs. Depending on molecular structure, ionic strength, temperature, and pH, these amphiphilic compounds and their combinations have the propensity to adopt lamellar and non-lamellar liquid crystalline phases on exposure to excess water (Nakano et al., 2002; de Campo et al., 2004; Yaghmur et al., 2005, 2017; Yaghmur and Glatter, 2009; Azmi et al., 2016; Gontsarik et al., 2016, 2018, 2019; Angelova et al., 2017). Thus, the lipolysis-triggered transition from emulsified oil droplets to lamellar or non-lamellar liquid crystalline interiors (Figure 3) was affected by the type of oil and attributed to the self-assembly of the digestion generated amphiphilic lipids in the lipolysis medium. The evolved scattering curves resembled those typically reported for liposomes, cubosomes, hexosomes, amongst others (Nakano et al., 2002; de Campo et al., 2004; Yaghmur et al., 2005; Yaghmur and Glatter, 2009; Azmi et al., 2016; Angelova et al., 2017; Gontsarik et al., 2018). Taking into account the suggested important role of these produced self-assembled nanostructures in facilitating the delivery and distribution of nutritional compounds throughout the body (Salentinig et al., 2011, 2015a, 2017; Salentinig, 2019; Vithani et al., 2019), there is a growing interest in exploring the implications for future development of advanced functional food and drug nanocarriers (Azmi et al., 2015; Angelova et al., 2017; Shao et al., 2018; Bor et al., 2019; Yaghmur, 2019).

**Figure 5:**
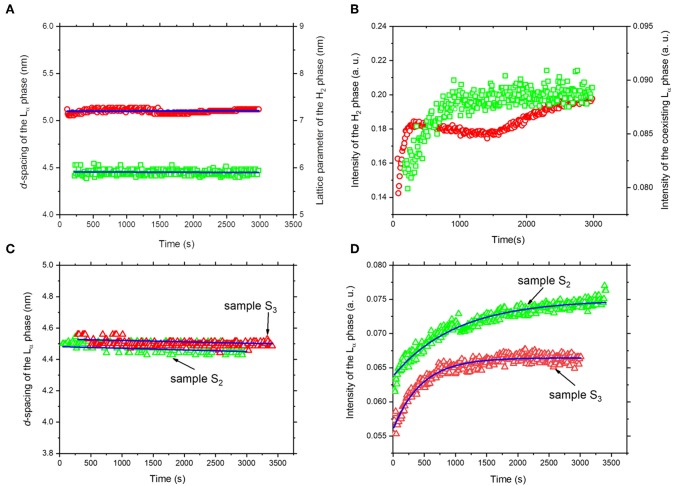
The calculated lattice constants **(A)** and temporal evolution **(B)** of the detected inverse hexagonal (H_2_) and lamellar (L_α_) phases during lipolysis of sample S_1_ (sodium caseinate-stabilized KO emulsion). The H_2_ and L_α_ phases are represented by open red circles and green squares, respectively. For samples S_2_ and S_3_, the calculated lattice constant **(C)** and temporal evolution **(D)** of the detected lamellar (L_α_) phase during lipolysis are represented by open red and green triangles, respectively. In **(D)**, the solid blue line shows the best single exponential fit to the data. In **(A,C)**, the lattice constants of the liquid crystalline phases were calculated from the SAXS profiles presented in Figure 2.

Referring again to Figures 3A, 4A, the fast molecular re-distribution and self-assembly of the generated amphiphilic lipolysis products in the emulsified KO droplets led first to the appearance of the H_2_ phase. After about 80 s, the first order reflection (100) of this phase started to be visible as a very weak peak at *q*-value of about 1.01 nm^−1^ (Figure 4A). Its intensity increased with time of digestion and the two additional characteristic (110) and (200) reflections started to be evolved at *q*-values of about 1.74 and 2.01 nm^−1^, respectively, after about 280 s of lipolysis. At this lipolysis time, the first order reflection of the coexisting L_α_ phase started also to appear at *q* value of about 1.40 nm^−l^. Its identification was based on the appearance of very weak third characteristic reflection after 2,980 s of lipolysis at *q*-value of about 4.20 nm^−1^. Based on the position of the first order reflection that was clear to identify, the second order peak position at *q*-value (~2.80 nm^−1^) was calculated and marked in Figure 4. As shown in Figure 5A, the lattice parameter and *d*-spacing of the H_2_ and L_α_ phases were about 7.21 ± 0.04 and 4.50 ± 0.10 nm, respectively, and were almost not affected with increasing the lipolysis time up to 2,980 s. In this H_2_/L_α_ phase coexistence regime, the H_2_ phase became more ordered and developed with increasing lipolysis time as indicated from the time evolution of the intensity of its first order diffraction peak (Figure 5B). However, less time was required for the coexisting L_α_ phase to become fully developed as the intensity of its first order diffraction peak was increased up to ~910 s and then reached plateau with increasing further the lipolysis time (Figure 5B).

To compare the effect of *ω-3* PUFA-containing oil type on the lipolysis-triggered evolving behavior of lyotropic liquid crystalline phases in the emulsified emulsion droplets, two additional sets of time-resolved SAXS experiments were carried out on AO emulsions stabilized by using sodium caseinate (sample S_2_) and citrem (sample S_3_). As presented in Table 1, AO exists similar to FO in the form of triacylglycerols; whereas most of *ω-3* PUFAs as mentioned above are bound to phospholipids in KO. This significant difference between these two oils in lipid composition, phospholipid content, molecular structure of *ω-3* PUFAs (triacylglycerols as compared to phospholipids) was reflected in the SAXS profiles presented in Figure 3 for the three investigated emulsions under identical experimental conditions. For AO emulsions (samples S_2_ and S_3_, Table 2), it was evident from the SAXS profiles presented in Figures 3B,C that the associated changes in the lipid composition of the emulsified AO during lipolysis led to the appearance of a neat L_α_ phase. Replacing sodium caseinate with citrem did not have a significant effect on the kinetics of structure formation. However, it is important to bear in mind that the replacement of sodium caseinate and citrem by non-digestible polymeric stabilizers could lead to a significant decrease in the digestibility of these emulsions due to restrictions on lipase accessibility to the oil localized in the droplets' interiors. In a previous study, Gudipati et al. (2010) reported on the important role of the stabilizer adsorption pattern in modulating the digestibility of FO emulsions, and showed that the replacement of citrem by non-digestible polymeric stabilizers is associated with a decrease in the digestion rate.

The experimental findings in Figures 5C,D provide more insight into the evolved neat L_α_ phase in both samples. Similar to sodium caseinate-stabilized KO emulsion (Figure 5A), the *d*-spacing of the L_α_ phase in samples S_2_ and S_3_ was 4.48 ± 0.02 and 4.52 ± 0.10 nm, respectively, and was almost not affected within the lipolysis periods of 3,010 and 3,410 s, respectively (Figure 5C). Thus, the replacement of sodium caseinate by citrem did not induce any significant change in the *d*-spacing of the L_α_ phase. These results indicate that there is no significant incorporation of sodium caseinate or citrem into AO during the emulsion preparation and digestion, and are in contrast with our previous findings on the significant effect of this emulsifier (citrem) on the structural features of lamellar and non-lamellar liquid crystalline nanoparticles based on soybean phospholipid, *ω-3* PUFA monoglycerides, or monoolein (Hedegaard et al., 2013; Azmi et al., 2015, 2016; Wibroe et al., 2015; Yaghmur et al., 2017; Prajapati et al., 2018; Shao et al., 2018). The time evolution of the first order reflection of the L_α_ phase and the single exponential function used to fit the SAXS data (full blue line) are presented in Figure 5D. The formation of the developed L_α_ phase in samples S_2_ and S_3_ was accomplished after ~1,500 and 1,250 s, respectively. It is interesting that the oil type did not have any significant influence on the *d*-spacing of the L_α_ phase in the three investigated samples (Figures 5A,C). The reason for this similarity in the *d*-spacing of the L_α_ phase in emulsified KO and AO droplets stabilized either by sodium caseinate or citrem is unclear but could be attributed to the presence of relatively similar lipid composition (mainly FFAs) in the lipolysis-triggered L_α_ phase in these samples. It is clear that the effects of lipid composition of KO and AO, and the concentration and type of *ω-3* PUFA-containing lipolysis products on modulating their digestibility and the associated dynamic structural features warrant further investigation.

## Conclusions

The lipolysis-induced dynamic phase behavior in emulsions prepared using two different *ω-3* PUFA-containing oils, in which *ω-3* PUFAs exist either in the form of triacylglycerols (AO) or phospholipids (KO), was compared. *In situ* SAXS combined with an *in vitro* lipolysis model depicted the dynamic structural features occurring during lipolysis of three KO and AO emulsions, stabilized using either sodium caseinate or citrem. It was evident from SAXS findings that the *ω-3* PUFA-containing oil type and its lipid composition play an important role in modulating the structural properties of the lipolysis-induced phases. The adsorption of lipase to the outer surfaces of emulsion droplets that was followed by the digestion of the emulsified marine oils (KO and AO) triggered fast molecular lipid re-arrangements and incorporation of the lipolysis products into their cores. This leads to the generation of internal biphasic H_2_/L_α_ features and neat L_α_ phases in the digested KO and AO emulsion droplets, respectively. It is interesting that the stabilizer type (sodium caseinate vs. citrem) and composition of KO and AO did not influence the detected L_α_ phase that had a *d*-spacing of about 4.5 nm in the three investigated emulsions. Table 3 presents our experimental findings. The structural parameters of lyotropic liquid crystalline phases identified in digested KO and AO emulsions are given and compared to those previously reported in digested human breast milk and mayonnaise.

**Table 3:** Structural parameters of lyotropic lamellar and non-lamellar liquid crystalline phases identified in digested KO and AO emulsions at 37°C and selected published findings for digested human breast milk and mayonnaise as determined from SAXS analysis.

**Investigated system**	**Space group**	**Lattice parameter, *a* (nm)**	**References**
KO-in-water emulsion	H_2_	7.20	Present study
	L_α_	4.50 ± 0.10	
Sodium caseinate -stabilized AO-in-water emulsion	L_α_	4.48 ± 0.02	Present study
Citrem-stabilized AO-in-water emulsion	L_α_	4.52 ± 0.10	Present study
Human breast milk	*Fd3m*	~15.3–15.7	(Salentinig et al., 2015a)
	H_2_	6.50	(Salentinig et al., 2015a)
	*Im3m*	20.85	(Salentinig et al., 2015a)
Mayonnaise	*Fd3m*	~16.50–17.50	(Salentinig et al., 2017)
	H_2_	~5.70–7.40	
	*Pn3m*	~24.20–27.90	

KO and AO are attractive for the development of functional food and drug nanocarriers owing to the beneficial health effects of *ω-3* PUFAs, and the important role of lipase-mediated lipolysis in generating nano-self-assemblies. These structures may facilitate the transport and delivery of nutrients and bioactive food additives throughout the body. The presented findings may have implications for functional food delivery applications with controllable digestibility, through an improved understanding of the digestion mechanism and the role of the evolved self-assembled nanostructures in modulating digestibility.

## Data Availability Statement

The datasets generated for this study are available on request to the corresponding author.

## Author Contributions

AY and SS: conceptualization and design of experiments. SL prepared and characterized the krill and astaxanthin oil-in-water emulsions. SA, GB, MG, AY, and SS performed the SAXS experiments. SL and SA analyzed and processed the SAXS data under the supervision of SS and AY. SL, SA, and AY wrote the article. MG, GB, and SS reviewed and edited the article.

### Conflict of Interest

The authors declare that the research was conducted in the absence of any commercial or financial relationships that could be construed as a potential conflict of interest.

## Abbreviations

AO: astaxanthin
DLS: dynamic light scattering
DHA, docosahexaenoic acid (22:6: n-3)
EPA, eicosapentaenoic acid (20:5: n-3)
FDA: American Food and Drug Administration
FFAs: free fatty acids
FO: fish oil
KO: krill oil
GRAS: Generally Recognized as Safe
*ω-3* PUFAs: omega-3 polyunsaturated fatty acids
PBS: phosphate buffer
PC: phosphatidylcholine
PDI: polydispersity index
SAXS: small angle X-ray scattering.
